# Effects of Emodepside on Single-Channel Properties of Onchocerca volvulus SLO-1A (BK) Potassium Channels

**DOI:** 10.21203/rs.3.rs-7206784/v1

**Published:** 2025-08-04

**Authors:** Charity Nya Njeshi, Shivani Choudhary, Mark Andrew McHugh, Sudhanva Srinivas Kashyap, Alan Patrick Robertson, Richard John Martin

**Affiliations:** Iowa State University; Iowa State University; Iowa State University; Iowa State University; Iowa State University; Iowa State University

**Keywords:** onchocerciasis, SLO-1K, electrophysiology, single-channel currents, emodepside, verruculogen

## Abstract

**Background:**

Control of onchocerciasis (river blindness of humans due to infection with the filarial nematode, *Onchocerca volvulus*) remains a challenge because of the lack of effective adulticides and vaccines. Emodepside is a broad-spectrum veterinary anthelmintic that has been found to inhibit nematode muscle activity by activating their tetrameric SLO-1K channels. Emodepside has adulticidal activity and is being trialed for onchocerciasis treatment, but the molecular mode of action of emodepside is still being elucidated. Here we examine the single-channel currents of *Ovo*-SLO-1A, a SLO-1K splice variant from *O. volvulus*, and explore how emodepside modulates the dynamics of the opening of the channel.

**Methods:**

*Ovo*-SLO-1A was expressed in HEK 293 cells, and patch clamp electrophysiology techniques were used to record currents. Single-channel currents were recorded in a symmetrical 132 mM K^+^ solution to determine the main open-state channel conductance. Emodepside’s effects were tested at 0.3 μM and 1.0 μM.

**Results:**

*Ovo*-SLO-1A had a main open-state conductance of 110 ± 3 pS and frequent flickering sub-conductance states. The presence of the flickering sub-conductance states suggests that there is limited cooperativity between the tetrameric channel subunits required for opening to the main open-state. Emodepside increased mean current amplitudes. Emodepside also increased open-burst times, and open probability. Verruculogen (1 μM) inhibited channel opening in the presence or absence of emodepside.

**Conclusion:**

This study successfully expressed *Ovo*-SLO-1A in HEK 293 cells, measured the conductance of the main open-state and detected the presence of sub-conductance states, and flickering openings. The increased amplitudes of the single-channel currents, open-burst times and open probabilities provide insights into how emodepside increases Slo-1K currents and illustrate dynamic actions of emodepside on *Ovo*-SLO-1A.

## Introduction

Onchocerciasis, caused by *Onchocerca volvulus*, is the second most important cause of infectious blindness after trachoma [1, 2]. Approximately 21 million people are infected and 246 million live in areas where onchocerciasis is present [3, 4]. Unfortunately, treatment options are limited due to the absence of an effective adulticide or vaccine, and further challenged by the development of drug resistance and reduced efficacy of treatment [5, 6]. This has underscored the need for new therapeutic strategies to combat onchocerciasis.

A promising avenue for novel drug development lies in the pharmacological characterization of ion channels as drug targets. Among these are the SLO-1K channels, also known as big conductance calcium-activated and voltage-sensitive potassium channels or BK channels. They are critical for membrane repolarization in excitable cells [7]. The SLO-1K channels are formed by a tetrameric assembly of alpha subunits, encoded by the single *slo* gene [8]. In nematodes, the channels are found in their body wall muscles and neurons, where pharmacological activation leads to loss of motility [9] and feeding by pharyngeal pumping [10]. Thus, modulating SLO-1K channel activity would lead to dysregulation of important cellular processes necessary for the organism’s survival, including movement and feeding.

In *O. volvulus*, studies have identified five isoforms of the SLO-1K channel (isoforms a, b, c, d and f), which result from alternative splicing [11, 12]. Selective pharmacological modulation of these channels presents a promising strategy for treatment and prevention of onchocerciasis. Emodepside, a cyclooctadepsipeptide, has been shown to activate SLO-1K channels [9]. It is a veterinary anthelmintic that has demonstrated anti-filarial activity against larvae and adults of different filarial nematodes [13, 14]. Its safety and efficacy for the treatment of nematode infections in cats and dogs has led to clinical trials for the treatment of human onchocerciasis.

Two-electrode voltage-clamp studies on *Xenopus* oocytes expressing SLO-1K channels have shown that emodepside activates the isoforms of the SLO-1K channel present in *O. volvulus* [12]. To date the effects of emodepside on the dynamics of the opening of SLO-1K channels have not been described. Wei et al., [15] compared single-channel properties and currents of the mouse, mSLO-1, and *Drosophila*, dSLO-1 channels. They found that the channels have a transmembrane core that determines the channel open-times, the voltage-dependence and channel sub-conductance [15]. In this study, we expressed the *Ovo*-SLO-1A channel of *O. volvulus* in the HEK 293 cell expression system and investigated how emodepside alters its single-channel properties. We found that the channel had flickering sub-conductance states, and that emodepside increased the mean channel amplitude and open probability (*P*_*open*_). We also tested verruculogen, a BK channel inhibitor, and found that it inhibited channel opening in the presence or absence of emodepside.

## Methods

### Cloning

*O. volvulus slo-1a* was synthesized by Life Technologies GeneArt (BioPark Regensburg, Germany). Primers for the gene were designed with sequences flanking the pcDNA 3.1-T2A-GFP expression vector that included the restriction site (Nhe1). PCR was conducted on the *O. volvulus slo-1a*. Subsequently, the amplicon was separated on a 1% Agarose SYBR Safe gel, purified using NucleoSpin Gel and PCR Cleanup Kit (Macherey-Nagel, Allentown, PA, USA) and then cloned into the pcDNA 3.1-T2A-GFP vector by using the Infusion HD Cloning Kit (Takara Bio USA, Inc, San Jose, CA, USA) according to the manufacturer’s protocols. Once cloned, the plasmids were verified by sequencing.

### HEK 293 Cell culture and transfection

HEK 293 cells were maintained in DMEM (GIBCO, ThermoFisher Scientific Waltham MA, USA) with 10% FBS (GIBCO ThermoFisher Scientific Waltham MA, USA) and 1% penicillin/streptomycin antibiotics in a cell culture flask (CORNING, AZ, USA) at 37°C in a humidified environment with 5% CO_2_. Media was changed every two days. Once cells reached 80–90% confluency, they were sub-cultured to smaller 50ml culture flasks. Transfections were carried out in these smaller flasks when the cells reached 80–90% confluency, following standard protocol. Briefly, the medium was removed and replaced with Opti-MEM^®^ reduced serum medium (GIBCO ThermoFisher Scientific Waltham MA, USA) 1 hour prior to transfection. The pcDNA 3.1-*Ovo-slo1a*-T2A-GFP construct was transiently transfected into HEK 293 cells using the Lipofectamine^™^ 2000 (Invitrogen, Carlsbad, CA, USA) protocol. On separate flasks, transfections with empty plasmid (pcDNA 3.1-T2A-GFP) for control recordings were done in the same ratios as the transfections with the gene of interest. The GFP in the plasmid construct served as a transfection marker. Cells were incubated with transfection mixtures for 6–12 hours, after which the medium was replaced with DMEM/10% FBS and incubated at 29°C, 5% CO_2_. Cells were detached from the petri dish using trypsin after 24–48 hours, seeded on coverslips coated with poly-D-lysine and incubated for 2 hours at 37°C, 5% CO_2_ prior to imagery and patch recordings. The fluorescent image was captured at 20X using the BZ 800 viewer from a Keyence digital microscope, equipped with a blue-to-green GFP filter box (EX 470/40 DM 495 BA 525/50, Osaka, Japan).

### Electrophysiology

#### Recording Conditions

Borosilicate glass electrodes (1.50-mm OD Clark Electromedical Instruments, UK) were pulled with a Narishige PC-100 Vertical puller (Narishige, Tokyo, Japan). The pipette tips were coated with Sylgard and fire polished using a Narishige Micro Forge MF-900 (Narishige CO., Ltd, Tokyo, Japan). Pipette resistances between 4 and 12 MΩ were used for recordings. Coverslips were transferred to a cell chamber 2 hours after plating and mounted on the stage of a stabilized inverted epifluorescence microscope (Nikon ECLIPSE TE 2000-U, Tokyo, Japan). Fluorescent cells were identified for patching by observing at 20X and 40X using the SOLA light engine (Lumencor light engine Beaverton, OR, USA) equipped with a band pass blue to green GFP filter. Patch-clamp recordings were done in the whole-cell, and inside-out configurations. All experiments were conducted at room temperature.

For whole cell experiments, the drug solution was delivered to the chamber under gravity feed through solenoid valves controlled using a VC-8 Eight-Channel Valve Controller (Warner Instruments, Hamden, CT, USA). For inside-out recordings, drugs were manually added to the bath and the cells exposed for 5 to 30 mins (depending on the drug) to allow enough time for diffusion from the point of application to the channels in the patches. All experiments where we observed channel rundown or membrane breakdown were excluded. After each experiment where drugs were applied to the bath, the coverslip in the experimental chamber was replaced with another containing a new sample of cells.

To investigate the drug effects on the single-channel properties of *Ovo*-SLO-1A, we recorded events in the absence of drug for approximately 2 mins and then added the first drug (either emodepside or verruculogen). For experiments where both drugs were added, once the effect of the first drug added was observed, the second drug was added and recordings continued for 20 mins. While we added emodepside to patches with initial low activity, we added verruculogen to patches with high initial channel activity so the effect of each drug could be easily identified.

#### Recording Solutions

For experiments to determine the conductance of the channel, we used inside-out patches with solutions that were symmetrical on both sides of the membrane (6 mM NaCl, 132 mM KCl, 1.2 mM MgCl_2_, 1 mM CaCl_2_, 11 mM Glucose and 10 mM HEPES, pH 7.4). For whole-cell experiments, the pipette solution contained 140 mM KCl, 1.2 mM MgCl_2_, 5.4 mM CaCl_2,_ 5 mM EGTA, 2 mM dipotassium ATP and 10 mM HEPES, pH 7.2, giving a free [Ca^2+^] of 100 μM [16]; and 137 mM NaCl, 5.9 mM KCl, 1.2 mM MgCl_2_, 2.2 mM CaCl_2_, 14 mM Glucose and 10 mM HEPES, pH 7.4 in the bath. For investigation drug effects on inside-out patches we used solutions containing: 150 mM NaCl, 4 mM KCl, 2 mM MgCl_2_, 2 mM CaCl_2_ and 10 mM HEPES, pH 7.4 in the pipette; and 150 mM KCl, 9.77 mM CaCl_2,_ 10 mM EGTA and 10 mM HEPES, pH 7.2. The bath [Ca^2+^] was adjusted to be 100 μM [16] using the MAX CHELATOR program: https://somapp.ucdmc.ucdavis.edu/pharmacology/bers/maxchelator/CaMgATPEGTA-TS.htm).

#### Drugs

Emodepside and verruculogen were purchased from Advanced ChemBlock Inc (Hayward, CA, USA). Stock solutions of emodepside (3 mM) and verruculogen (10 mM) for inside-out recordings were prepared in dimethyl sulfoxide (DMSO) and then diluted in the recording solution (to 3 μM for emodepside and 10 μM for verruculogen) prior to experimentation. Thus, the final concentration of DMSO in the experimental solutions was kept below 0.1%. We added a small volume of the drugs to the bath and allowed approximately 15 minutes for equilibration so that the final concentration in the bath of emodepside was 0.3 μM and the final concentration of verruculogen was 1 μM. For the whole cell recording with emodepside, the stock solution was prepared at 1 mM and diluted in recording solution to 1 μM.

## Data Analysis

Data were collected using an Axopatch 200B amplifier (Molecular Devices, LLC. San Jose, CA, USA), filtered at 2 kHz with an 8-pole Bessel filter, and sampled at 10 kHz with a Digidata 1550B (Molecular Devices, LLC. San Jose, CA, USA); and the pCLAMP Software versions 10.7.0 and 11.1.0 (Molecular Devices, LLC. San Jose, CA, USA). Openings or closings shorter than 0.5 ms were not well resolved. To determine the main open-state channel conductance, currents were measured at four different membrane potentials (± 50 mV and ± 75 mV), in the symmetrical solutions with 132 mM K^+^. A current-voltage plot was generated, and the channel conductance was determined from the slope.

For whole-cell recordings, peak currents responses elicited by the drug were measured, from baseline current prior to drug addition, using Clampfit. GraphPad Prism 10.3.0 software (GraphPad Software Inc., San Diego, CA, USA) was used to generate histograms. Unpaired Student’s *t*-test to test for statistical significance was used. A *p* value < 0.05 was considered significant, and results were expressed as mean ± S.E.M.

Single-channel analysis (amplitudes, open burst-times and probability of being open) was performed using Clampfit 11.1.0 and Clampfit 11.7.0. For Fig. 2A, absolute maximum amplitudes were calculated while for Fig. 5, the mean current amplitude values and open burst-times were calculated from values generated over a defined recording segment in Clampfit. Data were sorted using Microsoft excel and histograms generated in Clampfit 11.1.0 and GraphPad Prism 10.3.3. Gaussian functions were fitted to describe and illustrate the presence of the sub-conductance in the presence of emodepside. The open burst-times were defined as a single opening, or groups of openings separated by a close period of ≥ 0.5 ms. Probability of being open was calculated using the formula:

$${Np}_{o}=\left(\sum_{1}^{L}{To}_{L}\right)/T$$

Where *N* is the number of channels in the patch, *L* is the number of channels open, *To*_*L*_ is the total time *L* channels are open, and *T* is the duration of the recording analyzed.

Burst-times were not normally distributed (Shapiro-Wilk test), so we used the Wilcoxon signed-rank test which is a non-parametric test to compare burst-times before and after the addition of emodepside. All error bars represent Standard Error of the Mean (SEM), and paired t-tests were utilized where appropriate. The burst-time distribution histograms ≥ 0.5 ms were fitted to a single exponential with the Clampfit software.

## Results

### Expression and Emodepside-Mediated Activation of *Ovo*-SLO-1A in HEK 293 Cells

**Figure 1:**
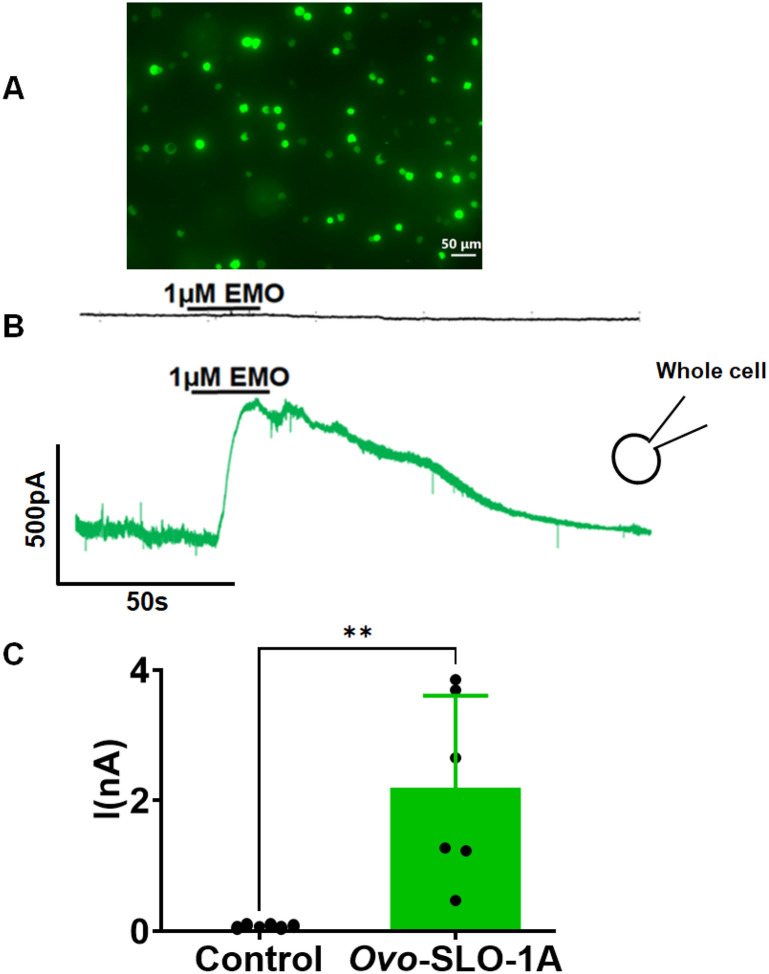
Expression of *Ovo*-SLO-1A in HEK 293 cells and activation by emodepside holding potential, +20mV. **A.** Fluorescent image showing expression of pcDNA 3.1(+) *Ovo slo-1a* T2A plasmid tagged with GFP. **B.** Representative traces: HEK 293 cells transfected with empty pcDNA 3.1(+) T2A GFP plasmid (black) and pcDNA 3.1(+) *Ovo slo-1a* T2A GFP plasmid (green); Insert of a whole cell configuration. **C.** Histogram showing current response to 1 μM emodepside in HEK 293 cells transfected with empty pcDNA 3.1(+) T2A GFP plasmid (control-black; Mean ± SEM; −0.01 ± 0.02 pA) and pcDNA 3.1(+) *Ovo slo-1a* T2A GFP plasmid (green; Mean ± SEM; 2.20 ± 0.58 pA); n = 6; *p* < 0.003; Scale bar 50 μm

Fig.1A shows a fluorescent image of HEK 293 cells transfected with pcDNA 3.1-*Ovo-slo1a*-T2A-GFP. The presence of green fluorescence indicated the probability of expression of the channel protein. We tested the effect of 1 μM emodepside at +20 mV using the whole-cell configuration. Emodepside induced outward currents in *Ovo-slo1a* transfected cells but not in controls (Fig. 1B). The difference in current amplitudes was statistically significant (Mean ± SEM; −0.01 ± 0.02 nA for control and 2.20 ± 0.58 nA for *Ovo-slo*-1a transfected HEK 293 cell; n = 6; *p* value < 0.0033), as illustrated in the histograms (Fig. 1C).

### Main Open-State Conductance of *Ovo*-SLO-1A

**Figure 2:**
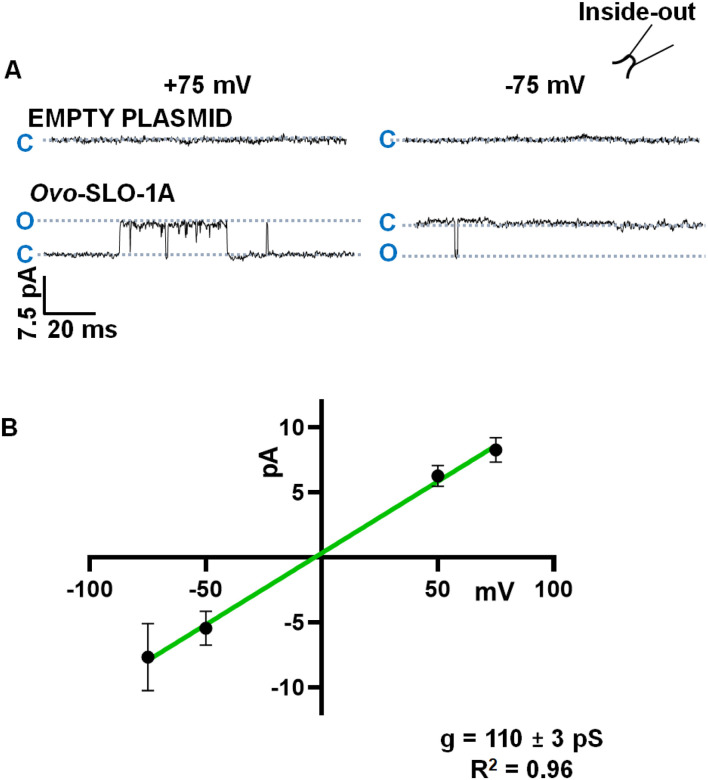
Full open-state Conductance of *Ovo*-SLO-1A expressed in HEK 293 cells. **A.** Representative traces from inside-out patches from HEK 293 cells transfected with empty pcDNA 3.1(+) T2A GFP plasmid (control - top) and pcDNA 3.1(+) *Ovo slo-1a* T2A GFP plasmid (bottom) at +75 mV and −75 mV; Insert of an inside-out configuration. **B.** Current-Voltage plot for recordings from HEK 293 cells transfected with pcDNA 3.1(+) *Ovo slo-1a* T2A GFP plasmid; conductance was 110 ± 3 pS, r^2^ = 0.96 (n = 6).

Representative current traces showing openings to the main open-state obtained from inside-out patches using symmetrical solutions on both sides of the patch containing 132 mM K^+^ held at +75 mV and −75 mV, are shown in Fig. 2A. Channel openings to the main open-state (seen as outward and inward currents) were observed in *Ovo-slo1a* transfected cells but not in control cells. A current-voltage (I-V) plot was generated using currents recorded from *Ovo-slo-1*a-transfected cells. The slope of this plot yielded a full open-state single-channel conductance of 110 ± 3 pS (n = 6) (Fig. 2B).

### Sub-conductance Levels Observed in *Ovo*-SLO1A

**Figure 3:**
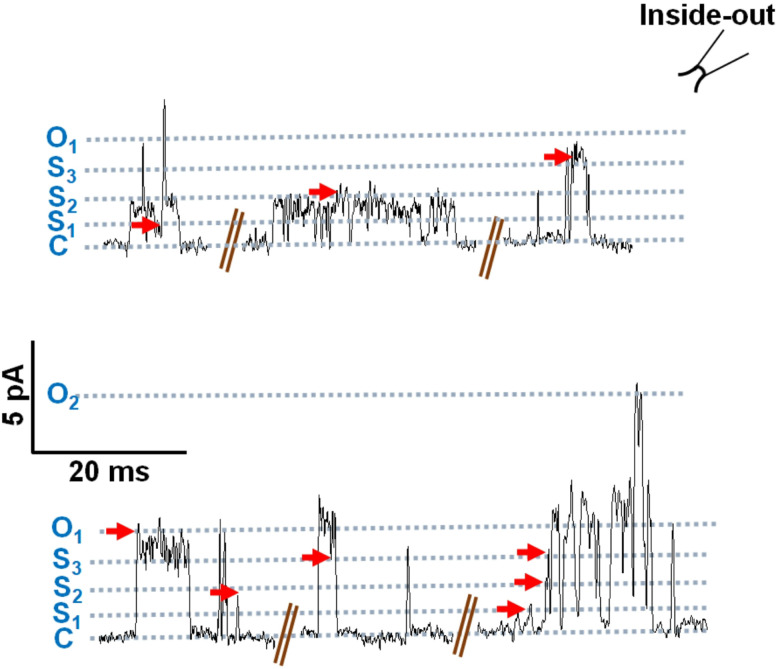
Sub-conductance behavior of the *Ovo*-SLO-1A channel. Representative traces for inside-out patch recordings from HEK 293 cells transfected with pcDNA 3.1(+) *Ovo slo-1a* T2A GFP, holding at +20 mV. Three sub-conductance states besides the main open-state and closed-state are shown.

Chapman and VanDongen [17] have pointed out the presence of short-lived sub-conductance states in addition to the main open-state of tetrameric K^+^ channels that are visited when the channel gate moves between the closed- and fully open-states. Similar sub-conductance behavior has been observed with some but not all BK channels [18,19]. These short-lived sub-conductance states give rise to a ‘flickering’ opening. The sub-conductance states are also observed with the mouse and *Drosophila* SLO-1K channels [20]. Current traces recorded from our inside-out patches from *Ovo*-SLO-1a transfected cells also revealed short-lived and sometimes longer-lived sub-conductance levels in addition to the main open-state. Fig. 3 illustrates the presence of three sub-conductance levels in addition to the closed and fully open state. The long-lived sub-conductance levels, were not limited by the time-resolution, 0.5 ms, of our recording system. We determined the mean of the single-channel current amplitudes for the recording by averaging the amplitudes of the openings to each of the sub-conductance levels and the opening to the main open-state.

Representative traces for inside-out patch recordings from HEK 293 cells transfected with pcDNA 3.1(+) *Ovo slo-1a* T2A GFP, holding at +20 mV. Three sub-conductance states besides the main open-state and closed-state are shown.

### Emodepside alters the single-channel properties of *Ovo*-SLO-1A

**Figure 4:**
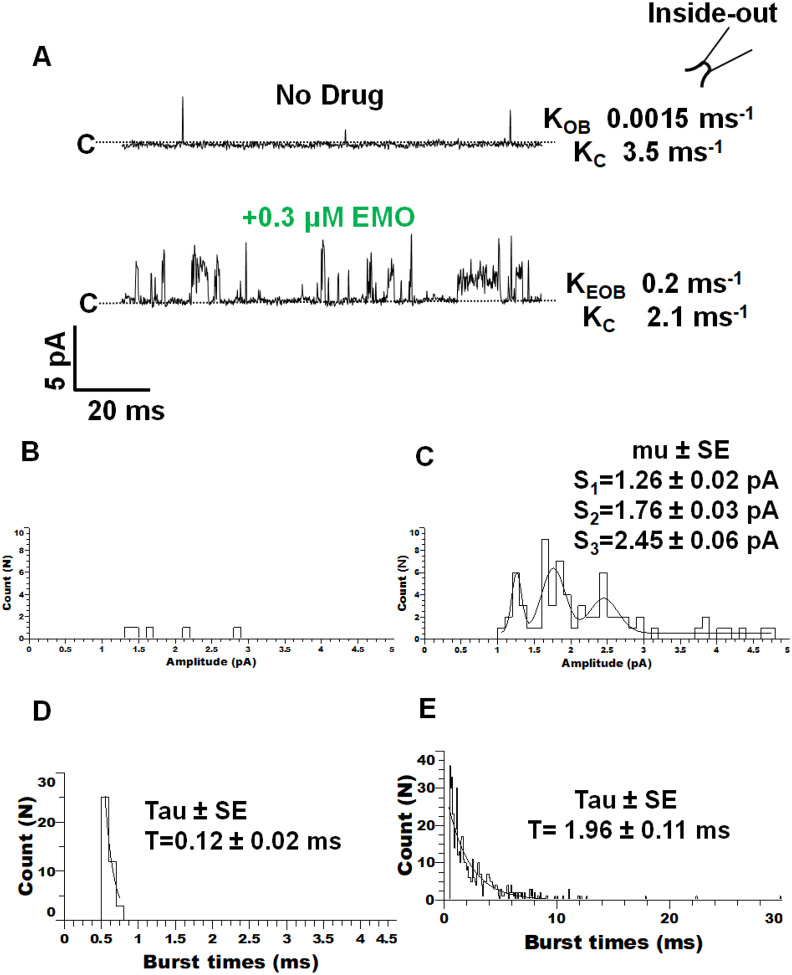
Effect of emodepside on the single-channel properties of *Ovo*-SLO-1A (holding, +20mV). **A.** Representative traces showing portion with no drug added (top; K_OB_ (burst-rate equilibrium) = 0.0015 ms^−1^ and K_C_ (burst closing-rate) = 3.45 ms^−1^ and 0.3 μM emodepside added (bottom; K_EOB_ (emodepside burst-rate) = 0.2 ms^−1^ and K_C_ (burst closing-rate equilibrium) = 2.12 ms^−1^). Insert of an inside-out configuration. **B.** Representative amplitude histogram without emodepside. **C.** Representative amplitude histogram for with emodepside, with the distribution fitted to three Gaussian functions to sub-conductance openings (S_1_: 1.26 ± 0.02 pA; S_2_: 1.76 ± 0.03 pA; S_3_: 2.45 ± 0.06 pA). **D.** Representative burst-times histogram for without emodepside with the distribution ≥ 0.5 ms fitted to a single exponential with a time-constant, of 0.12 ms **E.** Representative burst-times histogram following the addition of 0.3 μM emodepside with the distribution fitted to a single exponential with time a constant of 1.96 ms.

Application of emodepside induced clear changes to the single-channel currents of *Ovo*-SLO-1A, in our inside-out recordings. Our data revealed an increase in channel activity produced by emodepside application. Fig. 4A shows a patch before the application of 0.3 μM emodepside that had a burst opening-rate, K_OB_, of 0.0015 ms^−1^ and a burst closing-rate, K_C_, of 3.45 ms^−1^. Here, the addition of 0.3 μM emodepside increased the burst opening-rate, K_EOB_, to 0.2 ms^−1^ and slowed the burst closing-rate, K_C_, to 2.12 ms^−1^. With the longer open/burst times we observed that sub-conductance levels were more frequent and distinguishable with emodepside and that emodepside produced some open-channel currents that had bigger amplitudes, Fig. 4B and C. To illustrate the presence of the sub-conductance states in the amplitude histogram, we fitted Fig. 4C with the sum of 3 Gaussian distributions that had current peaks at 1.26pA. 1.76pA and 2.45pA. When we analyzed the burst durations, we found that bursts were longer in the presence of emodepside due to the appearance of longer bursts of openings (Fig. 4D and E).

### Averaged effects of emodepside and channels from different patches

**Figure 5:**
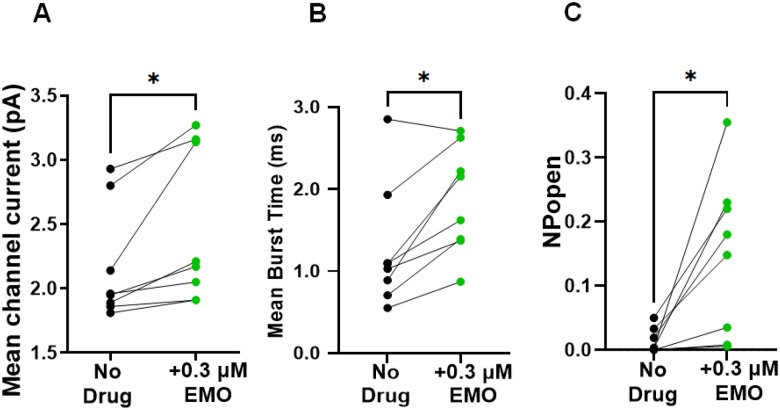
Effect of emodepside on *Ovo*-SLO-1A channel kinetics. **A.** Mean current before (Mean ± SEM; 2.17 ± 0.16 pA) and after adding 0.3 μM emodepside (Mean ± SEM; 2.48 ± 0.21 pA); *p* = 0.03 (paired *t*-test). **B.** Mean burst-time before (Mean ± SEM; 1.27 ± 0.27 ms) and after adding 0.3 μM emodepside (Mean ± SEM; 1.87 ± 0.23 ms); *p* = 0.02 (Wilcoxon signed-rank test). **C.** *NP*_*open*_ before (Mean ± SEM; 0.01 ± 0.01) and after adding 0.3 μM emodepside (Mean ± SEM; 0.15 ± 0.04); *p* = 0.02 (paired *t*-test), n = 8 for all experiments.

To look at population effects, we measured the effects of 0.3 μM emodepside at +20 mV on mean current amplitudes, log mean burst-times, and *P*_*open*_ in eight inside-out patches. Emodepside significantly increased the mean current amplitudes from the control of 2.17 ± 0.16 pA to 2.48 ± 0.21 pA (Means ± SEMs, n = 8; *p* = 0.03; Fig. 5A). The mean burst-time also increased from 1.3 ± 0.3 ms to 1.9 ± 0.2 ms (Means ± SEMs; n = 8; *p* = 0.02; Fig. 5B). The probability of the channel being open, *P*_*open*_, significantly increased from 0.01 ± 0.01 to 0.15 ± 0.04 (Means ± SEMs; n = 8; *p* = 0.02; Fig. 5C).

### Inhibition of *Ovo*-SLO-1A by verruculogen

**Figure 6:**
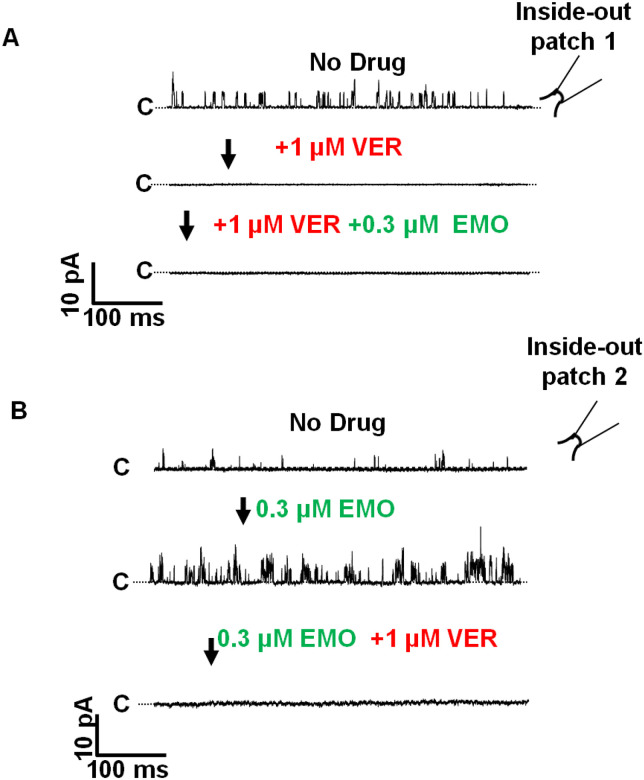
Verruculogen inhibits *Ovo*-SLO-1A activity in inside-out patches (holding at +20mV). **A.** Sections of sequential (arrow) recordings of channel openings (upward) and inhibition of openings (no openings). Top: no drug added. Middle: 1 μM verruculogen added. Bottom: a combination of 1 μM verruculogen and 0.3 μM emodepside. n = 6. Insert of an inside-out configuration. **B.** Sections of sequential (arrow) recordings of channel openings (upward) and inhibition of openings (no openings). Top: no drug added (top). Middle: 0.3 μM emodepside. Bottom: combination of 0.3 μM emodepside and 1 μM verruculogen. n = 5.

We conducted additional experiments using 1 μM verruculogen, a known inhibitor of SLO-1K channels. Application of verruculogen (in the absence of emodepside, n=6) inhibited channel activity, and this inhibition was not reversed by addition of 0.3 μM emodepside (Fig. 6A). The effect of 1 μM verruculogen in the presence of 0.3 μM emodepside (5 experiments) was also to inhibit channel activity (Fig. 6B).

## Discussion

### Effects of emodepside on SLO-1 K channels

Emodepside, is a cyclooctadepsipeptide and a broad-spectrum veterinary anthelmintic used for the treatment of dog and cat gastro-intestinal nematode infections. It is known to have effects on filarial nematodes and is undergoing clinical trials for the treatment of onchocerciasis (river blindness). Although emodepside has been found to increase filarial SLO-1K currents [9,12] under voltage-clamp when expressed in *Xenopus* oocytes, its effects at the single-channel level has not been investigated.

It has been proposed previously that PF1022A, a depsipeptide analogue of emodepside, by itself transports K^+^ ions across membranes by forming channel pores in the membrane [21,22]. We looked for and did not observe channel currents in HEK 293 cells without SLO-1K channels (empty plasmids in our control experiments) when emodepside was present in the patch. Our observations are not consistent with emodepside forming a channel pore in the membrane. Our observations show that emodepside alters the single-channel properties of *Ovo*-SLO-1A, increasing the frequency and amplitude of its open states.

Verruculogen, a BK channel antagonist, inhibited currents in *Ovo-slo*-1a transfected cells as it does with the *Drosophila* SLO-1 channels [23]. Raisch et al. [23] reported that the emodepside and verruculogen binding sites overlap within the channel, suggesting that binding of one may hinder or limit the binding of the other. Whole-cell patch-clamp studies on a *C. elegans* SLO-1K homolog reported reduced verruculogen inhibition when channels were pre-saturated with emodepside [7]. In our experiments, when emodepside was applied before verruculogen, inhibition still occurred. This suggests that emodepside is not irreversibly locked in position, keeping the channel open but may instead move in and out of its binding pocket.

In this study, we expressed *Ovo-slo*-1A in HEK 293 cells and observed the effects of application of emodepside to inside-out patches on their single-channel properties. We found that the *Onchocerca* SLO-1A K channels had ‘flickery’ openings and were able to identify three sub-conductance states and a full open-state. The full open-state had a single-channel conductance of 110pS. The conductance of the main open-state is similar to other SLO-1K channels which have large single-channel conductances in the range of 100 to 300pS [24].

Cryo-EM experiments on the *Drosophila melanogaster* SLO-1 K channel have observed that emodepside binds in the S6 pocket of the channel, across the central symmetry axis beneath the selectivity filter, as a stable ionophore [23]. We observed that emodepside increased *P*_*open*_, mean channel amplitudes, and burst-times in our inside-out patches of Ovo-SLO-1A but did not produce long, stable amplitude open-states. Our observations support the idea that emodepside helps stabilize the open-states of *Ovo*-SLO-1A but does not lock it open in a single, long-lasting open-state, but allows oscillations between the four open-states and the closed-state.

### Sub-conductances, flickery openings and *Ovo*-SLO-1A alanine 350

**Figure 7:**
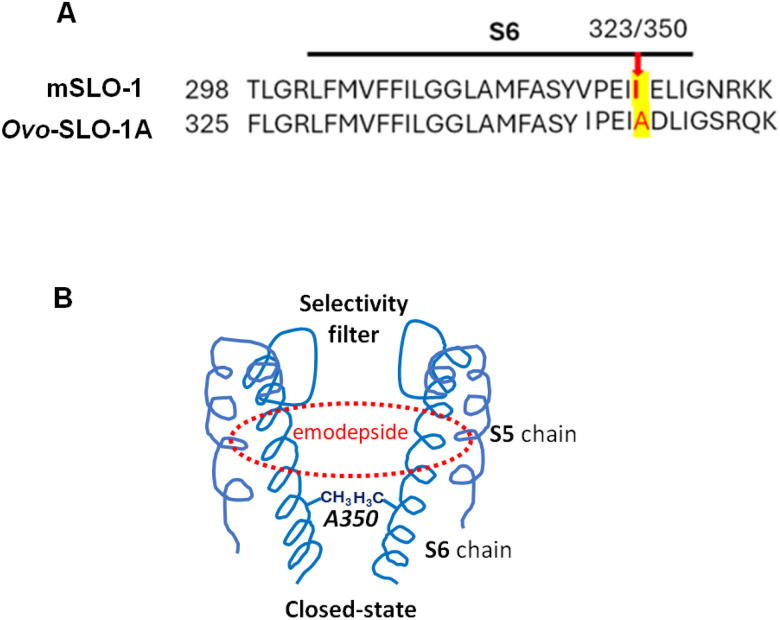
The S6 amino-acid sequence of mouse SLO-1 and *Ovo*-SLO-1A and location of A350 and binding region of emodepside. **A.:** Note the presence of isoleucine 323 in the mouse SLO-1 and the presence of alanine 350 in *Ovo*-SLO-1A. The alanine’s decreased hydrophobicity can explain the reduction in the cooperativity between the 4 subunits of the SLO-1A channel, the presence of sub-conductance states and flickery channel opening. **B.** Diagram of the location of alanine at position 350 on the S6 region of two opposing subunits of the *Ovo*-SLO-1A channel. The narrowing region near A350 forms a hydrophobic barrier near the cytoplasmic pore entrance that stabilizes the closed state. The A350 is smaller and less hydrophobic than isoleucine, reducing the opening cooperativity between the channel subunits, which leads to the sub-conductance states and flickering opening [17–20]. Also shown is the binding region of emodepside, see reference [22].

The mouse, mSLO-1, I323 position is equivalent to position 350 of *Ovo*-SLO-1A, Fig. 7A. To better understand the regulation of opening and sub-conductance mechanisms of SLO-1K channels, Guo et al., [20] studied the single-channel currents of mouse mSLO-1 I323 mutants which have ‘flickery’ openings with sub-conductances levels. They found that: 1) the hydrophobicity at the mSLO-1 323 position affects the appearance of sub-conductance levels of the channel; and 2) because mSLO-1 323 is the last pore-lining amino-acid of the S6 region, it also regulates gating [20]. The side chains of the position mSLO-1 I323 form the narrowest hydrophobic gateway of the channel at the cytoplasmic pore entrance, Fig. 7B, and holds the four channel protein subunits together to increase the cooperativity of the channel subunits. Decreasing the hydrophobicity of I323 with a shorter side chain amino acid like alanine made the interactions between the subunits weaker and less cooperative and led to the sub-conductance states and flickery openings (shorter open-/ burst-times).

Alanine is found at the equivalent position in all *Ovo*-*slo*-1 splice variants and in other filaria including *Bugia malayi* and *Dirofilaria immitis* as well as in *Caenorhabditis elegans* splice variants. The presence of the *Ovo*-SLO-1A alanine at position 350 may explain the flickery openings and sub-conductances of the *Ovo*-SLO-1A channel currents that are not seen in mammalian BK channels. We observed flickering sub-conductance states and sometimes longer conductance states that have been interpreted to be due to the additive effects of individual conformations each of the tetrameric subunits contributing to form the open-state of the SLO-1K channel [17–20].

### Effects of emodepside on *Ovo*-SLO-1A channels

Our results provide insights into the gating properties of *Ovo*-SLO-1A channels and how emodepside alters the gating properties to increase potassium current that leads to its anthelmintic effects. Emodepside increased mean single-channel current amplitudes, *P*_*open*_ and burst-times to increase the total potassium membrane current. An increase in the number of bigger currents and full openings is consistent with emodepside binding in the S6 pocket of the channel [23] and increasing cooperativity between the four channel subunits to increase openings to the full open-state. However, the persistent presence sub conductance levels, suggests incomplete cooperativity. Other factors such as the positioning of the subunit may be needed to achieve full cooperativity even in the presence of emodepside [25]. The longer burst-times observed in the presence of emodepside shows that emodepside also shifts the equilibrium towards the open-states.

## Conclusion

This study reveals modulatory effects of emodepside on single-channel currents of *Ovo*-SLO-1A and channel subunit cooperativity. It demonstrates the dynamic features of the effects of emodepside characterized by increased open rates and decreased close rates, increased mean current amplitude, burst-times and *P*_*open*_.

## Data Availability

Data supporting the main conclusions of this study are included in the manuscript.
